# Supplementary material for: Effects of Outdoor and Household Air Pollution on Hand Grip Strength in a Longitudinal Study of Rural Beijing Adults
Source: Int J Environ Res Public Health. 2025 Aug 16;22(8):1283. doi: 10.3390/ijerph22081283 (PMC12386705; doi:10.3390/ijerph22081283)
Supplement: Supplementary file 1 [file ijerph-22-01283-s001.zip › ijerph-3685345-supplementary.pdf]

Supplemental Materials

The 24-hour personal exposure to $PM_{2.5}$ concentration measurements

The 24-hour personal exposure to $PM_{2.5}$ concentrations are included in multiple imputation as predictors of household $PM_{2.5}$. To measure personal exposure to $PM_{2.5}$, 50% of participants were randomly selected to wear a personal exposure monitor (PEMs, Apex Pro; Casella, UK) or an ultrasonic personal aerosol samplers (UPAS, Access Sensor Technologies, Fort Collins, CO, USA) for 24 hours to collect a filter-based $PM_{2.5}$ sample in each wave, including most of the households which measured household $PM_{2.5}$. Details on the measurement and estimation of personal $PM_{2.5}$ can be found elsewhere [33].

Table S1. Number of missingness and proportions in the dataset used for multiple imputation.

Variable	Number missing	Proportion in dataset (%)
**Participant ID**	0	0
**Study wave**	0	0
**Household ID**	0	0
**Village ID**	0	0
**County ID**	0	0
**District id**	0	0
**Time implemented clean heating policy**	0	0
**Whether implement clean heating policy this year**	0	0
**Gender**	0	0
**Age**	0	0
**Wintertime outdoor temperature (Jan 15 – March 15)**	0	0
**Wintertime outdoor dew point (Jan 15 – March 15)**	0	0
**Wintertime outdoor $PM_{2.5}$ (Jan 15 – March 15)**	0	0
**Maximum grip strength**	0	0
**Type of fuel used for heating**	5	0.23
**Diabetes**	7	0.32
**Chronic obstructive pulmonary disease**	7	0.32
**Asthma**	7	0.32
**Tuberculous**	7	0.32
**Chronic hepatitis**	7	0.32
**Cirrhosis**	7	0.32
**Kidney disease**	7	0.32
**Rheumatoid arthritis**	7	0.32
**Tobacco smoke**	7	0.32

**Body mass index**	7	0.32
**Number of cigarettes smoked by the participant per day**	8	0.37
**Frequency of drinking**	8	0.37
**Frequency of exercising**	8	0.37
**Frequency of farming**	8	0.37
**Self-reported health status**	8	0.37
**Hypertension**	8	0.37
**Heart disease**	8	0.37
**Stroke**	8	0.37
**Feeling about wind**	8	0.37
**Hospitalized during the past 12 months**	11	0.51
**Type of fuel used for cooking**	16	0.74
**Indoor temperature measured during home visit**	18	0.83
**Highest education obtained**	22	1.01
**Marital status**	27	1.24
**Heating area in the home**	27	1.24
**Current occupation**	28	1.29
**Number of household occupants in winter**	29	1.33
**Space heating duration per day**	29	1.33
**Waist circumference**	39	1.79
**Type of fuel used for heating and boiling water**	56	2.57
**Estimated payment for electricity in the previous winter**	70	3.22
**Asset-based wealth index quartile**	74	3.40
**Presence of insulation in the home**	780	35.85
**24-hour personal exposure to $PM_{2.5}$**	1201	55.19
Received monitor (n=1121) but no measurements	146	-

Not sampled for personal exposure measurements	1055	-
**Wintertime household $PM_{2.5}$ (Jan 15 – March 15)**	1769	81.3
Received monitor (n=476) but no measurements	69	-
Not sampled for wintertime household $PM_{2.5}$ measurements	1700	-

Note: Variables listed in this table were all included in multiple imputation model, but only a sub-set were included in the statistical analysis for this study.

Table S2 Comparisons of the descriptive statistics for observed (non-imputed) versus observed plus imputed household $PM_{2.5}$.

	Min	Median	Max	Geometric mean	Geometric SD	Mean	SD
**Observed**	3.04	55.11	431.03	57.52	2.27	79.75	69.55
**Imputed plus Observed**	3.04	61.31	431.03	60.75	2.41	87.63	78.01

Table S3. Selected participant characteristics.

	Wave 1 (winter 2018-2019, n=648)		Wave 2 (winter 2019-2020, n=775)		Wave 4 (winter 2021-2022, n=753)	
	Mean (± SD or %)	# of missing (%)	Mean (± SD or %)	# of missing (%)	Mean (± SD or %)	# of missing (%)
**Age, years**	60.4 (8.9)	-	61.4 (8.9)	-	63.4 (8.9)	-
**Sex, female**	395 (61.0)	-	462 (59.6)	-	462 (61.4)	-
**Marital status**		21 (3.2)		6 (0.8)		-
Married	557 (86.0)		674 (87.0)		642 (85.3)	
Divorced or separated	15 (2.3)		14 (1.8)		11 (1.5)	
Widowed	51 (7.9)		71 (9.2)		93 (12.4)	
Never Married	4 (0.6)		10 (1.3)		7 (0.9)	
**Highest education**		21 (3.2)		1 (0.1)		-
No school	77 (11.9)		86 (11.1)		91 (12.1)	
Primary school	479 (73.9)		586 (75.6)		565 (75.0)	
Secondary or high school	66 (10.2)		90 (11.6)		86 (11.4)	
Higher education	5 (0.8)		12 (1.5)		11 (1.5)	
**Current occupation**		21 (3.2)		7 (0.9)		-
Agriculture	417 (64.4)		473 (61.0)		372 (49.4)	
Other manual labor[a]	7 (1.1)		12 (1.5)		20 (2.7)	
Non-manual labor[b]	21 (3.2)		55 (7.1)		85 (11.3)	
Unemployed	139 (21.5)		157 (20.3)		264 (35.1)	
Others	43 (6.6)		71 (9.2)		12 (1.6)	
**Tobacco smoke**		5 (0.8)		2 (0.3)		-
No exposure to tobacco smoke	134 (20.7)		153 (19.7)		125 (16.6)	
Never smoker lived with smoker	260 (40.1)		315 (40.6)		331 (44.0)	
Former Smoker	82 (12.7)		112 (14.5)		115 (15.3)	
Current smoker	167 (25.8)		193 (24.9)		182 (24.2)	
**Frequency of drinking**		5 (0.8)		3 (0.4)		-
Never	341 (52.6)		377 (48.6)		379 (50.3)	

Occasional (≤ 3 times a month)	133 (20.5)		172 (22.2)		164 (21.8)	
Regular (≤ 5 times a week)	49 (7.6)		64 (8.3)		59 (7.8)	
Everyday	120 (18.5)		159 (20.5)		151 (20.1)	
**Frequency of farming**		5 (0.8)		3 (0.4)		-
Never	226 (34.9)		316 (40.8)		336 (44.6)	
Occasional (≤ 3 times a month)	208 (32.1)		229 (29.5)		216 (28.7)	
Regular (≤ 5 times a week)	125 (19.3)		156 (20.1)		128 (17.0)	
Everyday	84 (13)		71 (9.2)		73 (9.7)	
**Frequency of exercising**		5 (0.8)		3 (0.4)		-
Never	107 (16.5)		182 (23.5)		185 (24.6)	
Occasional (≤ 2 days a week)	100 (15.4)		107 (13.8)		98 (13.0)	
Regular (≤ 5 days a week)	70 (10.8)		81 (10.5)		84 (11.2)	
Everyday	366 (56.5)		402 (51.9)		386 (51.3)	
**Self-reported health**		5 (0.8)		3 (0.4)		-
Poor	244 (37.7)		272 (35.1)		272 (36.1)	
Fair	261 (40.3)		351 (45.3)		321 (42.6)	
Good	117 (18.1)		130 (16.8)		126 (16.7)	
Excellent	21 (3.2)		19 (2.5)		34 (4.5)	
**Asset-based wealth index**		26 (4.0)		33 (4.3)		15 (2.0)
Bottom quartile (poorest)	168 (25.9)		190 (24.5)		157 (20.8)	
2nd quartile	163 (25.2)		202 (26.1)		155 (20.6)	
3rd quartile	151 (23.3)		162 (20.9)		228 (30.3)	
Top quartile (wealthiest)	140 (21.6)		188 (24.3)		198 (26.3)	
**BMI, kg/m^2**	26.0 (3.6)	1 (0.2)	25.7 (3.4)	-	26.3 (3.8)	6 (0.8)
**Waist circumference, cm**	86.6 (10.1)	11 (1.7)	87.2 (9.3)	6 (0.8)	91.2 (10.4)	22 (3.0)
**Wintertime $PM_{2.5}$[c], μg/m^3**						
Household (sampled)[d]	-	-	70.2 (2.1)	22 (10.0)	47.0 (2.4)	47 (18.9)
Outdoor	43.8 (1.4)	-	59.1 (1.3)	-	36.3 (1.3)	-
**Maximum grip strength, kg**	31.8 (10.1)	-	34.0 (10.0)	-	27.4 (9.8)	-

Note: Selected participant characteristics by quartile of average household $PM_{2.5}$ after multiple imputation are shown in Table 1 in the main text. [a] Other manual labor includes manufacturing, mining, and construction workers. [b] Non-manual labor includes government, technical, and professional service workers. [c] Geometric mean (geometric standard deviation) is presented for household and outdoor $PM_{2.5}$ considering their skewed distributions. [d] Household $PM_{2.5}$ was measured in 300 randomly selected households for the Beijing Household Energy Transition study in wave 2 and 4 (we didn't measured household $PM_{2.5}$ in wave 1). Among our study population, 227 and 249 of which were selected for household $PM_{2.5}$ measurement in wave 2 and 4, respectively, while 22 and 47 out of which have no measurements due to power shortages, sensor damage, or sensor data loss. Percentage of missingness is calculated based on participants selected for measuring household $PM_{2.5}$.

Table S4. Results from multivariable linear mixed effects regression models evaluating effects of wintertime (January 15 to March 15) household $PM_{2.5}$ (HOUSEHOLD) on maximum grip strength, with product terms between household $PM_{2.5}$ (both mean-centered and mean household $PM_{2.5}$) and sex, age, highest education, current occupation, tobacco smoke, drinking frequency, farming frequency, exercising frequency, and wealth index quartile. Results are shown as the point estimates and 95% confidence intervals (in kg).

	Change in grip strength (in kg) per 10 μg/m³ within-individual change	Change in grip strength (in kg) per 10 μg/m³ between-individual change	
	**Interaction[a]**	**Interaction**	**p-value**
**Sex (ref: female)**	0 (-0.10, 0.10)	-0.19 (-0.39, 0.02)	0.17
**Age[b]**	0 (0, 0.01)	0 (-0.01, 0.01)	<0.01
**Highest education (Ref: No School)**			0.99
Primary school	0.01 (-0.17, 0.19)	-0.05 (-0.29, 0.20)	
Secondary or high school	0.05 (-0.18, 0.29)	-0.11 (-0.46, 0.24)	
Higher education	-0.09 (-1.33, 1.15)	-0.2 (-1.32, 0.92)	
**Occupation (Ref: Agriculture)**			1.00
Other manual labor[c]	0.04 (-0.40, 0.49)	-0.01 (-0.61, 0.60)	
Non-manual labor[d]	0.04 (-0.26, 0.34)	0.04 (-0.23, 0.31)	
Unemployed	-0.02 (-0.14, 0.11)	0.05 (-0.08, 0.18)	
Others	-0.08 (-0.35, 0.19)	-0.05 (-0.34, 0.24)	
**Frequency of drinking (Ref: Never)**			0.82
Occasional (≤ 3 times/month)	0.03 (-0.11, 0.17)	-0.04 (-0.21, 0.12)	

Regular (≤ 5 times/week)	0.01 (-0.20, 0.22)	-0.02 (-0.28, 0.23)	
Everyday	-0.04 (-0.17, 0.09)	-0.14 (-0.32, 0.05)	
**Frequency of farming (Ref: Never)**			0.99
Occasional (≤ 2 days/week)	0 (-0.14, 0.14)	0.02 (-0.12, 0.15)	
Regular (≤ 5 days/week)	0.04 (-0.14, 0.21)	0.06 (-0.09, 0.22)	
Everyday	-0.01 (-0.20, 0.19)	0.01 (-0.18, 0.20)	
**Frequency of exercising (Ref: Never)**			0.88
Occasional (≤ 2 days/week)	-0.02 (-0.23, 0.19)	0.07 (-0.11, 0.26)	
Regular (≤ 5 days/week)	-0.05 (-0.26, 0.16)	0.02 (-0.19, 0.24)	
Everyday	0.01 (-0.12, 0.13)	-0.05 (-0.19, 0.10)	
**Tobacco smoke (Ref: No exposure)**			0.86
Never smoker lived with smoker	0.03 (-0.16, 0.22)	0.16 (-0.17, 0.48)	
Former Smoker	0.04 (-0.19, 0.28)	0.15 (-0.19, 0.50)	
Current smoker	0.01 (-0.19, 0.20)	0.03 (-0.26, 0.32)	
**Wealth index (Ref: Bottom quartile (poorest))**			0.92
2nd quartile	0.04 (-0.15, 0.22)	0.05 (-0.11, 0.21)	
3rd quartile	0 (-0.15, 0.16)	0.01 (-0.15, 0.17)	
Top quartile (wealthiest)	0.02 (-0.16, 0.21)	-0.06 (-0.22, 0.10)	

Note: The results are from multivariable model adjusted for sex, age, marital status, highest education, current occupation, exposure to tobacco smoke, typical number of cigarettes consumed per day if reported to be current smoker, frequency of drinking, frequency of farming, exercise frequency, self-reported health status, waist circumference (with 2 degrees of freedom natural cubic spline), and asset-based wealth index quartile. F-test was used to compare the multivariable model with and without the interaction terms, showing the p-value.

[a] Interaction refers to the interaction term between exposure (de-mean or mean exposure) and the effect modifier.

[b] Age were centered at mean age of the entire study population (62 years).

[c] Other manual labor includes manufacturing, mining, and construction workers.

[d] Non-manual labor includes government, technical, and professional service workers.

Table S5. Results from multivariable linear mixed effects regression models evaluating effects of wintertime (January 15 to March 15) outdoor $PM_{2.5}$ (OUTDOOR) on maximum grip strength, with product terms between outdoor $PM_{2.5}$ (both mean-centered and mean outdoor $PM_{2.5}$) and sex, age, highest education, current occupation, tobacco smoke, drinking frequency, farming frequency, exercising frequency, and wealth index quartile. Results are shown as the point estimates and 95% confidence intervals (in kg).

	Change in grip strength (in kg) per 10 μg/m³ within-individual change	Change in grip strength (in kg) per 10 μg/m³ between-individual change	
	**Interaction[a]**	**Interaction**	**p-value**
**Sex (ref: female)**	0.21 (-0.12, 0.53)	0.51 (-0.05, 1.07)	0.10
**Age[b]**	0 (-0.02, 0.02)	0.06 (0.03, 0.10)	<0.01
**Highest education (Ref: No School)**			0.31
Primary school	-0.07 (-0.58, 0.45)	-0.47 (-1.20, 0.26)	
Secondary or high school	-0.14 (-0.83, 0.54)	-1.19 (-2.18, -0.19)	
Higher education	0.44 (-1.31, 2.18)	0.84 (-1.77, 3.45)	
**Occupation (Ref: Agriculture)**			0.67
Other manual labor[c]	0.32 (-0.97, 1.61)	0.46 (-0.86, 1.77)	
Non-manual labor[d]	-0.35 (-0.99, 0.29)	0.15 (-0.56, 0.87)	
Unemployed	-0.36 (-0.77, 0.04)	0.13 (-0.29, 0.55)	
Others	0.04 (-0.94, 1.02)	0.33 (-0.45, 1.11)	
**Frequency of drinking (Ref: Never)**			0.61
Occasional (≤ 3 times/month)	0.07 (-0.36, 0.51)	0.25 (-0.24, 0.75)	
Regular	0.67 (-0.04, 1.38)	0.03 (-0.72, 0.79)	

(≤ 5 times/week)			
Everyday	0.02 (-0.42, 0.46)	0.07 (-0.60, 0.74)	
**Frequency of farming (Ref: Never)**			0.03
Occasional (≤ 2 days/week)	0.20 (-0.23, 0.62)	-0.08 (-0.50, 0.33)	
Regular (≤ 5 days/week)	-0.39 (-0.88, 0.09)	0.48 (-0.04, 1.00)	
Everyday	-0.08 (-0.70, 0.54)	0.78 (0.12, 1.45)	
**Frequency of exercising (Ref: Never)**			0.61
Occasional (≤ 2 days/week)	-0.44 (-1.02, 0.14)	-0.34 (-0.89, 0.21)	
Regular (≤ 5 days/week)	0.14 (-0.55, 0.82)	-0.05 (-0.69, 0.60)	
Everyday	-0.14 (-0.56, 0.28)	-0.07 (-0.54, 0.39)	
**Tobacco smoke (Ref: No exposure)**			0.32
Never smoker lived with smoker	-0.08 (-0.54, 0.37)	-0.32 (-0.99, 0.35)	
Former Smoker	-0.02 (-0.59, 0.54)	0.62 (-0.22, 1.46)	
Current smoker	-0.07 (-0.58, 0.44)	0.11 (-0.67, 0.90)	
**Wealth index (Ref: Bottom quartile (poorest))**			0.45
2nd quartile	0.1 (-0.42, 0.61)	0.31 (-0.19, 0.82)	
3rd quartile	0.37 (-0.16, 0.91)	0.24 (-0.26, 0.73)	
Top quartile (wealthiest)	0.33 (-0.29, 0.94)	-0.13 (-0.59, 0.33)	

Note: The results are from multivariable model adjusted for sex, age, marital status, highest education, current occupation, exposure to tobacco smoke, typical number of cigarettes consumed per day if reported to be current smoker, frequency of drinking, frequency of farming, exercise frequency, self-reported health status, waist circumference (with 2 degrees of freedom natural cubic spline), and asset-based wealth index quartile. F-test was used to compare the multivariable model with and without the interaction terms, showing the p-value. [a] Interaction refers to the interaction term between exposure (de-mean or mean exposure) and the effect modifier. [b] Age were centered at mean age of the entire study population (62 years). [c] Other manual labor includes manufacturing, mining, and construction workers.
[d] Non-manual labor includes government, technical, and professional service workers.

Table S6. Effects of wintertime (January 15 to March 15) average household and outdoor $PM_{2.5}$ on maximum grip strength limited to waves with measured household $PM_{2.5}$ (i.e., wave 2 and wave 4), presented as point estimates and 95% confidence intervals (in kg).

	Change in grip strength (in kg) per 10 μg/m³ within-individual change[a]		Change in grip strength (in kg) per 10 μg/m³ between-individual change[a]	
	**Multiple imputation**	**Complete case**	**Multiple imputation**	**Complete case**
**Household $PM_{2.5}$**	0.11 (0.01, 0.21)	0.22 (0.04, 0.40)	-0.10 (-0.25, 0.04)	-0.10 (-0.34, 0.14)
**Household $PM_{2.5}$ further adjusted with outdoor $PM_{2.5}$**	-0.01 (-0.09, 0.07)	0.03 (-0.12, 0.18)	-0.12 (-0.27, 0.02)	-0.13 (-0.36, 0.10)
**Outdoor $PM_{2.5}$**	2.06 (1.86, 2.27)	2.12 (1.90, 2.34)	0.41 (0.07, 0.75)	0.30 (-0.05, 0.70)

Note: *Multiple imputation* presents estimates from imputed data in wave 2 and wave 4. *Complete case* presents estimates from observed (non-imputed) complete cases data in wave 2 and wave 4 (i.e., no missingness in exposure, outcome, and covariates include in the multivariable model). [a] Adjusted for sex, age, marital status, highest education, current occupation, exposure to tobacco smoke, typical number of cigarettes consumed per day if reported to be current smoker, frequency of drinking, frequency of farming, exercise frequency, self-reported health status, waist circumference (with 2 degrees of freedom natural cubic spline), and asset-based wealth index quartile.

Table S7. Sensitivity analysis results from multivariable mixed-effects regression models with individual-level random intercept evaluating the effects of household or outdoor $PM_{2.5}$ on maximum grip strength, showing the point estimates and 95% confidence intervals (in kg).

	Change in grip strength (in kg) per 10 μg/m³ within-individual change		Change in grip strength (in kg) per 10 μg/m³ between-individual change	
	**Household $PM_{2.5}$**	**Outdoor $PM_{2.5}$**	**Household $PM_{2.5}$**	**Outdoor $PM_{2.5}$**
**Main**	0.06 (-0.01, 0.12)	1.51 (1.35, 1.68)	-0.05 (-0.20, 0.08)	0.21 (-0.07, 0.48)
**Main + village**[a]	0.06 (-0.01, 0.12)	1.51 (1.35, 1.68)	-0.03 (-0.17, 0.10)	0.31 (-0.08, 0.69)
**Main + co-morbidity**[b]	0.05 (-0.01, 0.12)	1.51 (1.34, 1.67)	-0.06 (-0.20, 0.08)	0.18 (-0.09, 0.46)
**Main + temperature**[c]	0.05 (-0.01, 0.12)	1.53 (1.37, 1.70)	-0.05 (-0.19, 0.08)	0.24 (-0.04, 0.51)
**Main + clean energy policy implementation**[d]	0.04 (-0.02, 0.11)	-	-0.05 (-0.19, 0.08)	-
**Main exclude highest 3% household $PM_{2.5}$**[e]	0.08 (-0.01, 0.16)	-	-0.04 (-0.19, 0.11)	-
**Main exclude highest 5% household $PM_{2.5}$**[f]	0.08 (-0.02, 0.18)	-	-0.04 (-0.37, 0.13)	-
**Main exclude household $PM_{2.5}$ measured less than 2 weeks**[g]	0.06 (-0.01, 0.12)	-	-0.05 (-0.19, 0.08)	-
**Main fitted with data from wave 1 & 2 only**[h]	-	0.73 (0.47, 0.99)	-	-0.04 (-0.37, 0.30)
**Main fitted with outdoor $PM_{2.5}$ measured before grip strength measurement**[i]	-	1.14 (0.98, 1.30)	-	0.13 (-0.15, 0.42)

Note: The *Main* model was fitted with wintertime (January 15 to March 15) average household $PM_{2.5}$ or outdoor $PM_{2.5}$ as exposure and adjusted with sex, age, marital status, highest education, current occupation, exposure to tobacco smoke, typical number of cigarettes consumed per day if reported to be current smoker, frequency of drinking, frequency of farming, exercise frequency, self-reported health status, waist circumference (with 2 degrees of freedom natural cubic spline), and asset-based wealth index quartile, using multiple imputed data. The results are pooled based on Rubin's rule [38]. Unless noted, all models were fitted with multiple imputed dataset. [a] *Main + village* is *Main model* with an additional random intercept for the participant's village of residence. [b] *Main + co-morbidity* is *Main model* further adjusted with co-morbid conditions (i.e., number of conditions include: self-reported clinician-diagnosed high blood pressure, chronic obstructive pulmonary disease, tuberculosis, diabetes, asthma, chronic hepatitis, cirrhosis, kidney disease, rheumatoid arthritis, heart disease, stroke, and hospitalization in the past 12 months). [c] *Main + temperature* is *Main model* further adjusted with the indoor temperature measured during home visit. [d] *Main + clean energy policy implementation* is *Main model* further adjusted with a binary variable indicating whether participant's living village implemented a clean energy policy. [e]

Main exclude highest 3% household $PM_{2.5}$ is the main household $PM_{2.5}$ model fitted with multiple imputed data excluding those with the highest 3% household $PM_{2.5}$ (i.e., wintertime household $PM_{2.5}$ concentration higher than 262.83 μg/m^3). [f] *Main exclude highest 5% household $PM_{2.5}$* is the main household $PM_{2.5}$ model fitted with multiple imputed data excluding those with the highest 5% household $PM_{2.5}$ (i.e., wintertime household $PM_{2.5}$ concentration higher than 230.21 μg/m^3). [g] *Main exclude household $PM_{2.5}$ measured less than 2 weeks* is the main household $PM_{2.5}$ model fitted with multiple imputed data excluding those with household $PM_{2.5}$ measured less than 2 weeks (20% of 2-month study period) in wintertime (n = 25). [h] *Main fitted with data from wave 1 & 2 only* is a multivariable model fitted with observed (non-imputed) complete cases data from wave 1 (winter 2018-2019) and wave 2 (winter 2019-2020) only, with wintertime (January 15 to March 15) outdoor $PM_{2.5}$ as exposure adjusted with the same set of covariates as *Main* model. [i] *Main fitted with outdoor $PM_{2.5}$ measured before grip strength measurement* is a multivariable model fitted with average outdoor $PM_{2.5}$ measured before or on the day of the interview as exposure (measured days ranged from 1 to 65 days with an average of 22 days) with observed (non-imputed) complete cases data from winter 2018-2019, winter 2019-2020, and winter 2021-2022, adjusted with the same set of covariates as *Main* model.

Table S8. Sensitivity analysis results evaluating the effects of outdoor $PM_{2.5}$ on maximum grip strength, comparing using wintertime (January 15 to March 15) average outdoor $PM_{2.5}$, and 1-day lag, 7-day lag, or 30-day lag outdoor $PM_{2.5}$ as exposure, showing the point estimates and 95% confidence intervals (in kg).

Number of observation	Change in grip strength (in kg) per 10 μg/m³ within-individual change		Change in grip strength (in kg) per 10 μg/m³ between-individual change	
	**Use wintertime measurement[a]**	**Use 1-day lag measurement[b,c]**	**Use wintertime measurement**	**Use 1-day lag measurements**
1154	2.11 (1.89, 2.33)	0.71 (0.54, 0.88)	0.30 (-0.06, 0.66)	0.08 (-0.15, 0.31)
	**Use wintertime measurement[a]**	**Use 7-day lag measurement[d]**	**Use wintertime measurement**	**Use 7-day lag measurements**
1040	1.97 (1.75, 2.19)	1.30 (1.06, 1.54)	0.39 (0.02, 0.76)	0.03 (-0.08, 0.61)
	**Use wintertime measurement[a]**	**Use 30-day lag measurement[e]**	**Use wintertime measurement**	**Use 30-day lag measurements**
242	1.73 (1.41, 2.05)	2.15 (1.75, 2.56)	-0.23 (-0.21, 1.61)	-0.48 (-2.22, 1.26)

Note: All model were adjusted with sex, age, marital status, highest education, current occupation, exposure to tobacco smoke, typical number of cigarettes consumed per day if reported to be current smoker, frequency of drinking, frequency of farming, exercise frequency, self-reported health status, waist circumference (with 2 degrees of freedom natural cubic spline), and asset-based wealth index quartile. All models were fitted with observed (non-imputed) data from waves 2 and 4 only. This is because in wave 1, most of the outdoor air pollution were measured on the day or after grip strength measurement so we excluded wave 1 data for this analysis. Additionally, we ensured the comparison of the models with wintertime or lag measurement were conducted in the same population. [a] Multivariable mixed-effects regression models with individual-level random intercept, using wintertime (January 15 to March 15) average outdoor $PM_{2.5}$ as exposure.

[b] Multivariable mixed-effects regression models with individual-level random intercept, using outdoor $PM_{2.5}$ prior to grip strength measurement with different lag windows as exposure. [c] 1-day lag outdoor $PM_{2.5}$ measurements refer to the average outdoor $PM_{2.5}$

measured 1 day before and on the day of grip strength measurement. [d] 7-day lag outdoor $PM_{2.5}$ measurements refer to the average outdoor $PM_{2.5}$ measured 7 days before and on the day of grip strength measurement.
[e] 30-day lag outdoor $PM_{2.5}$ measurements refer to the average outdoor $PM_{2.5}$ measured 30 days before and on the day of grip strength measurement.

Table S9. Selected participant characteristics of participants with and without grip strength measurements at baseline.

	Participants with grip strength measurements (n=648 participants)		Participants without grip strength measurements (n=267 participants)		
	**Mean (± SD or %)**	**# of missing (%)**	**Mean (± SD or %)**	**# of missing (%)**	**P-value[a]**
**Age, years**	60.4 (8.9)	-	58.9 (10.0)	5 (1.8)	0.02
**Sex, female**	395 (61)		150 (56.2)	3 (1.1)	0.28
**Marital status**		21 (3.2)		9 (3.4)	0.97
Married	557 (86)		231 (86.5)		
Divorced or separated	15 (2.3)		6 (2.2)		
Widowed	51 (7.9)		20 (7.5)		
Never Married	4 (0.6)		1 (0.4)		
**Highest education**		21 (3.2)		9 (3.4)	0.06
No school	77 (11.9)		30 (11.2)		
Primary school	479 (73.9)		198 (74.2)		
Secondary or high school	66 (10.2)		22 (8.2)		
Higher education	5 (0.8)		8 (3)		
**Current occupation**		21 (3.2)		10 (3.7)	0.19
Agriculture	417 (64.4)		151 (56.6)		
Other manual labor[b]	7 (1.1)		4 (1.5)		
Non-manual labor[c]	21 (3.2)		15 (5.6)		
Unemployed	139 (21.5)		66 (24.7)		
Others	43 (6.6)		21 (7.9)		
**Tobacco smoke**		5 (0.8)		4 (1.5)	0.93
No exposure to tobacco smoke	134 (20.7)		54 (20.2)		
Never smoker lived with smoker	260 (40.1)		101 (37.8)		
Former Smoker	82 (12.7)		36 (13.5)		
Current smoker	167 (25.8)		72 (27)		

**Frequency of drinking**		5 (0.8)		4 (1.5)	0.30
Never	341 (52.6)		150 (56.2)		
Occasional (≤ 3 times a month)	133 (20.5)		49 (18.4)		
Regular (≤ 5 times a week)	49 (7.6)		12 (4.5)		
Everyday	120 (18.5)		52 (19.5)		
**Frequency of farming**		5 (0.8)		4 (1.5)	0.11
Never	226 (34.9)		104 (39)		
Occasional (≤ 3 times a month)	208 (32.1)		74 (27.7)		
Regular (≤ 5 times a week)	125 (19.3)		40 (15)		
Everyday	84 (13)		45 (16.9)		
**Frequency of exercising**		5 (0.8)		4 (1.5)	0.35
Never	107 (16.5)		55 (20.6)		
Occasional (≤ 2 days a week)	100 (15.4)		38 (14.2)		
Regular (≤ 5 days a week)	70 (10.8)		33 (12.4)		
Everyday	366 (56.5)		137 (51.3)		
**Self-reported health**		5 (0.8)		4 (1.5)	0.67
Poor	244 (37.7)		101 (37.8)		
Fair	261 (40.3)		111 (41.6)		
Good	117 (18.1)		40 (15)		
Excellent	21 (3.2)		11 (4.1)		
**Asset-based wealth index**		26 (4)		22 (8.2)	0.97
Bottom quartile (poorest)	168 (25.9)		65 (24.3)		
2nd quartile	163 (25.2)		61 (22.8)		
3rd quartile	151 (23.3)		61 (22.8)		
Top quartile (wealthiest)	140 (21.6)		58 (21.7)		
**BMI, kg/m^2**	26 (3.6)	1 (0.2)	26.9 (4.1)	170 (63.7)	0.02
**Waist circumference, cm**	86.6 (10.1)	11 (1.7)	88.5 (10.5)	174 (65.2)	0.08
**Wintertime outdoor $PM_{2.5}$[d], μg/m^3**	43.8 (1.4)	-	47.8 (1.4)	-	<0.01

Note: Participants without grip strength are participants recruited for Beijing Household Energy Transition study but excluded from this study due to lack of grip strength measurements. We didn't measured household $PM_{2.5}$ at baseline (wave 1). [a] Indicates p-value of

Wilcoxon rank sum test (for outdoor $PM_{2.5}$ only), two-sample t-test (for other continous variable) or chi-square test (for categorical variables). [b] Other manual labor includes manufacturing, mining, and construction workers. [c] Non-manual labor includes government, technical, and professional service workers. [d] Geometric mean (geometric standard deviation) is presented for household and outdoor $PM_{2.5}$ considering their skewed distributions.

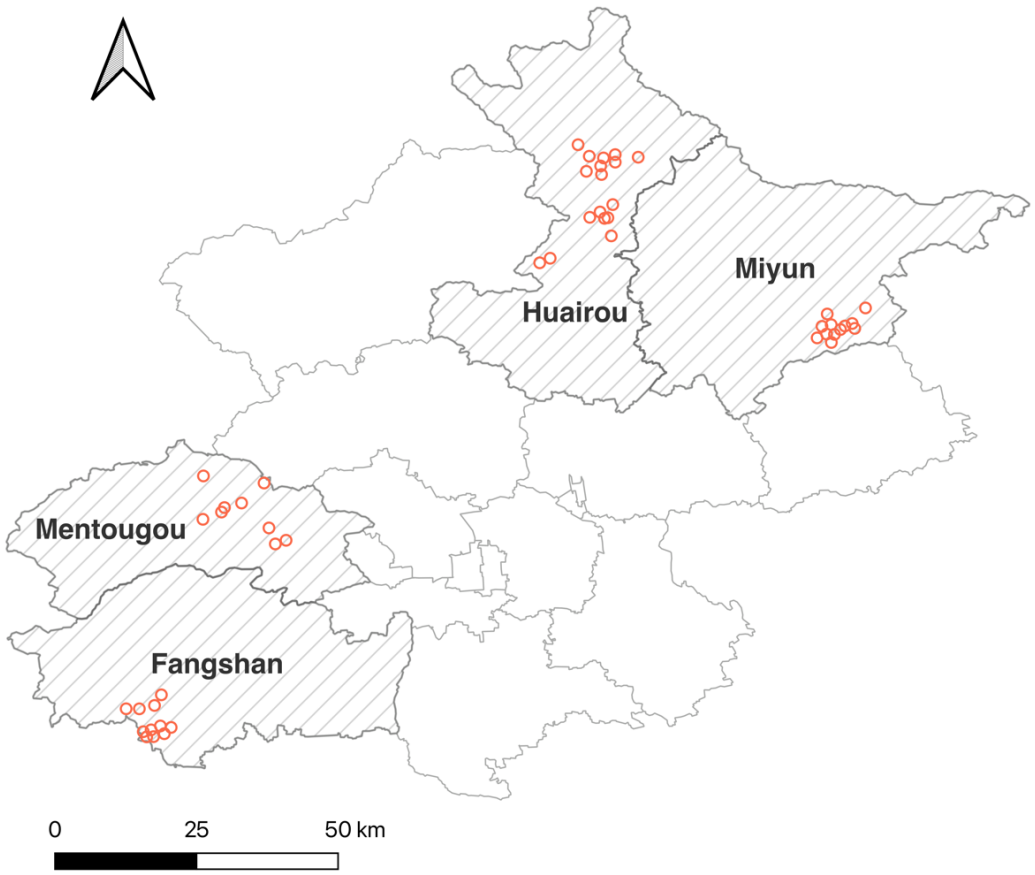

Figure S1. Map of study villages (n=50, indicated by red circles) and their districts in Beijing, China.

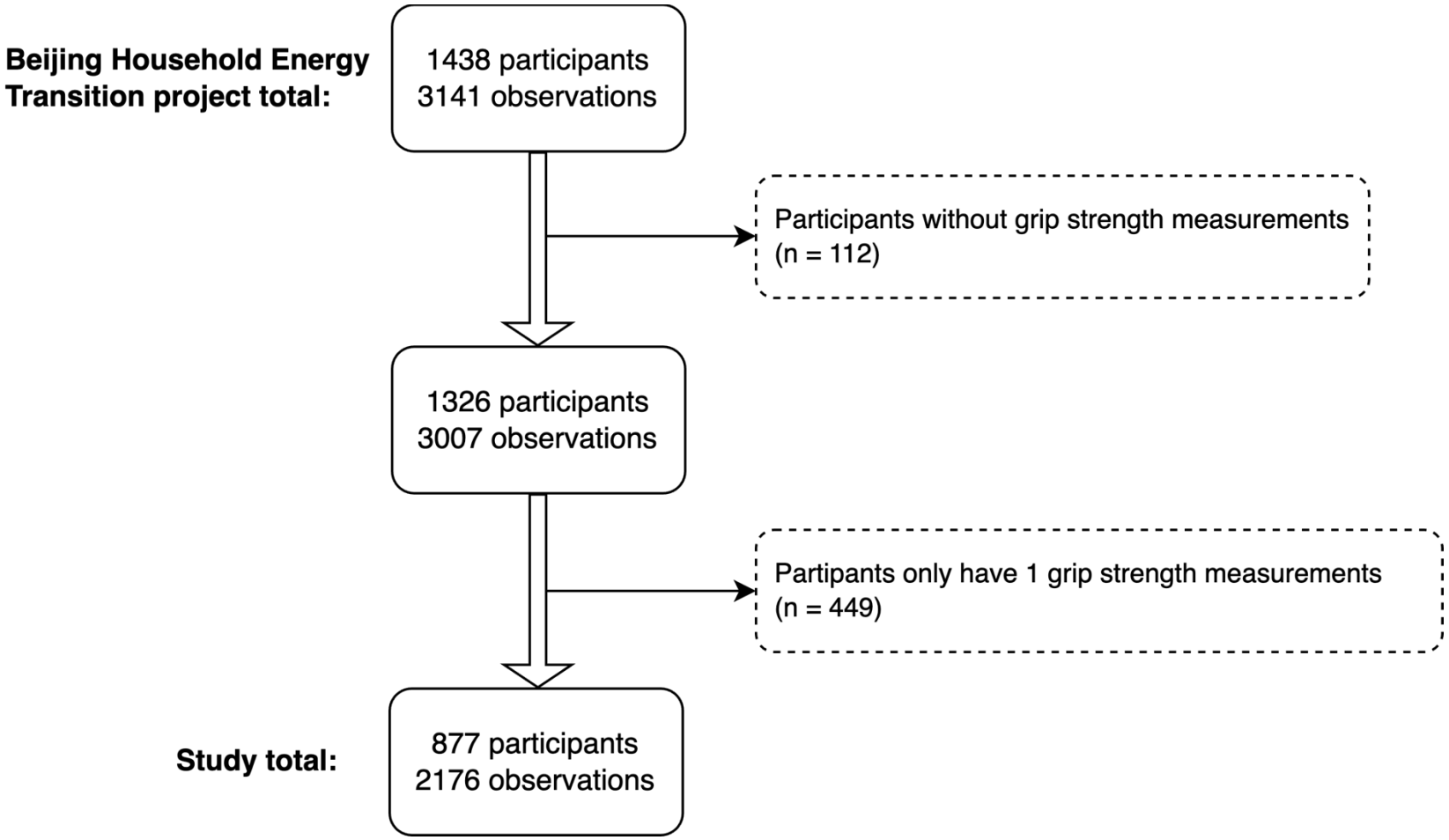

Figure S2. Flowchart of selection into this study.

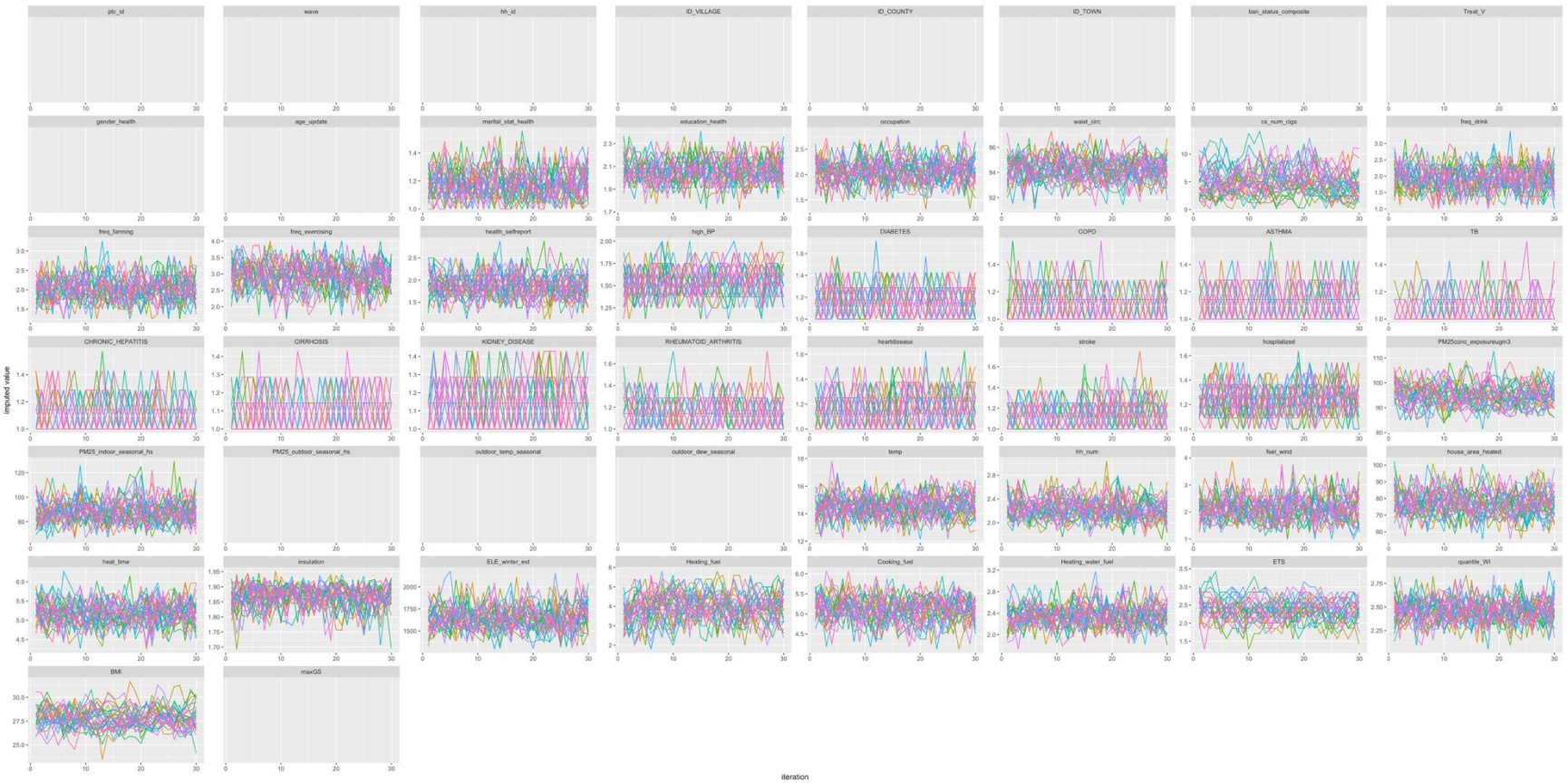

Figure S3. Trace plot of multiple imputation.

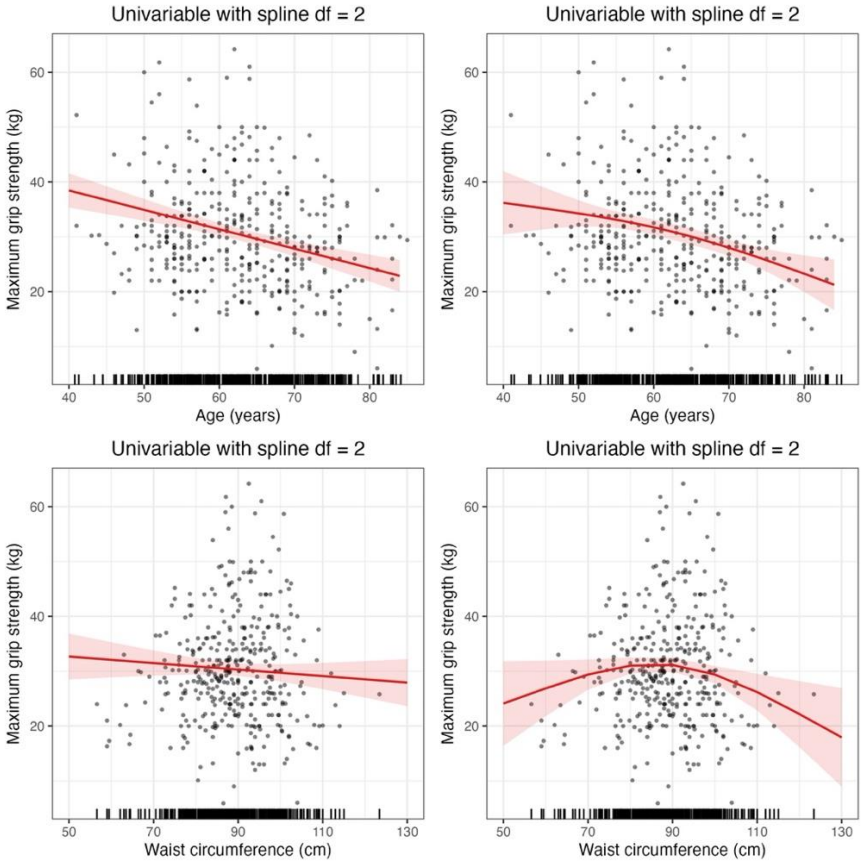

Figure S4. Univariable mixed effect model with individual level random effects, without and with natural cubic spline (2 degree of freedom), considering age and waist circumference as independent variable and maximum grip strength as dependent variable. Note: The model is based on observed (non-imputed) complete cases data of 389 observations from 265 observations (i.e., no missing in wintertime household and outdoor $PM_{2.5}$, sex, age, marital status, highest education, current occupation, exposure to tobacco smoke, typical number of cigarettes consumed per day if reported to be current smoker, frequency of drinking, frequency of farming, exercise frequency, self-reported health status, waist circumference, and asset-based wealth index quartile). Solid red line is showing point estimate with lighter red shade showing corresponding 95% confidence interval. Grey points represent observations.

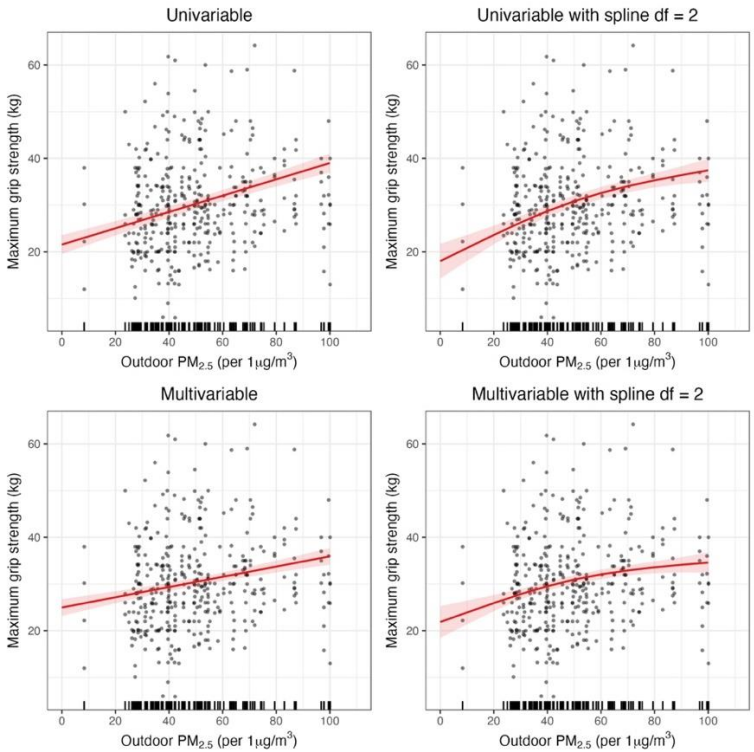

Figure S5. Univariable and multivariable mixed effect model with individual level random effects, without and with natural cubic spline (2 degree of freedom), evaluating wintertime outdoor $PM_{2.5}$ and maximum grip strength association. Note: The model is based on observed (non-imputed) complete cases data of 389 observations from 265 observations (i.e., no missing in wintertime household and outdoor $PM_{2.5}$, sex, age, marital status, highest education, current occupation, exposure to tobacco smoke, typical number of cigarettes consumed per day if reported to be current smoker, frequency of drinking, frequency of farming, exercise frequency, self-reported health status, waist circumference, and asset-based wealth index quartile). The multivariable model is adjusted with sex, age, marital status, highest education, current occupation, exposure to tobacco smoke, typical number of cigarettes consumed per day if reported to be current smoker, frequency of drinking, frequency of farming, exercise frequency, self-reported health status, waist circumference (with 2 degrees of freedom natural cubic spline), and asset-based wealth index quartile. Solid red line is showing point estimate with lighter red shade showing corresponding 95% confidence interval. Grey points represent observations.

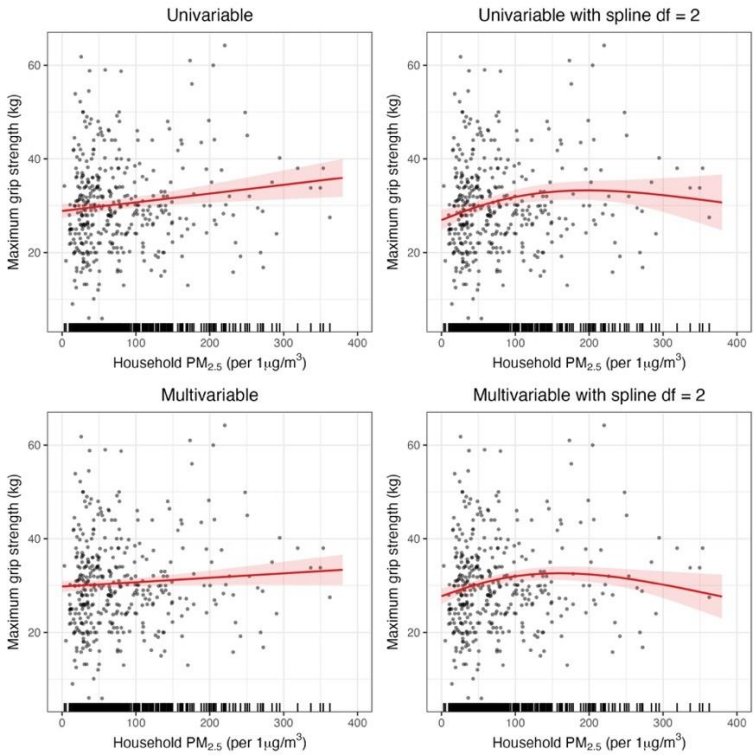

Figure S6. Univariable and multivariable mixed effect model with individual level random effects, without and with natural cubic spline (2 degree of freedom), evaluating wintertime household $PM_{2.5}$ and maximum grip strength association. Note: The model is based on observed (non-imputed) complete cases data of 389 observations from 265 observations (i.e., no missing in wintertime household and outdoor $PM_{2.5}$, sex, age, marital status, highest education, current occupation, exposure to tobacco smoke, typical number of cigarettes consumed per day if reported to be current smoker, frequency of drinking, frequency of farming, exercise frequency, self-reported health status, waist circumference, and asset-based wealth index quartile). The multivariable model is adjusted with sex, age, marital status, highest education, current occupation, exposure to tobacco smoke, typical number of cigarettes consumed per day if reported to be current smoker, frequency of drinking, frequency of farming, exercise frequency, self-reported health status, waist circumference (with 2 degrees of freedom natural cubic spline), and asset-based wealth index quartile. Solid red line is showing point estimate with lighter red shade showing corresponding 95% confidence interval. Grey points represent observations.

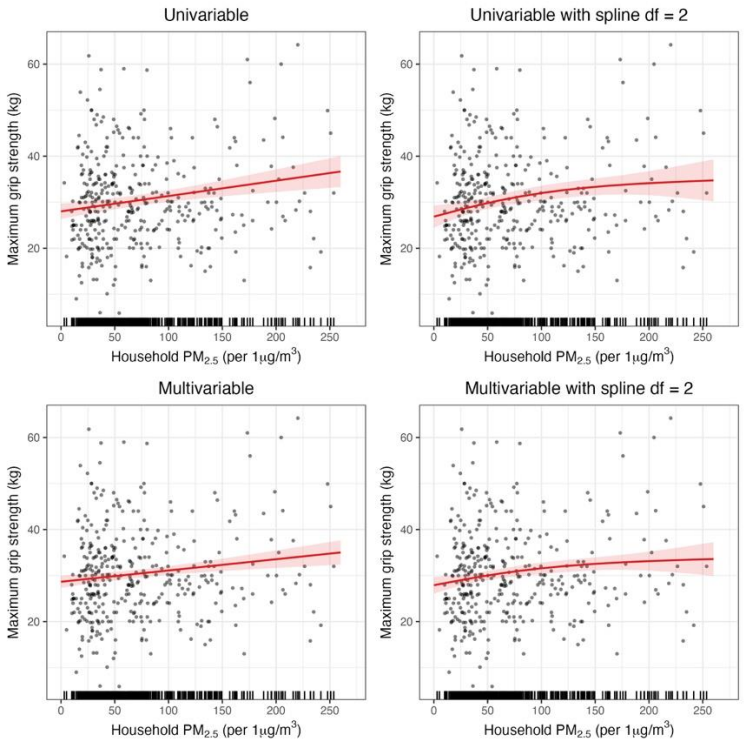

Figure S7. Univariable and multivariable mixed effect model with individual level random effects, without and with natural cubic spline (2 degree of freedom), evaluating wintertime household $PM_{2.5}$ and maximum grip strength association. Note: The model is based on observed (non-imputed) complete cases data (i.e., no missing in wintertime household and outdoor $PM_{2.5}$, sex, age, marital status, highest education, current occupation, exposure to tobacco smoke, typical number of cigarettes consumed per day if reported to be current smoker, frequency of drinking, frequency of farming, exercise frequency, self-reported health status, waist circumference, and asset-based wealth index quartile) excluding those with highest 3% of household $PM_{2.5}$, resulting in 377 observations from 259 observations). The multivariable model is adjusted with sex, age, marital status, highest education, current occupation, exposure to tobacco smoke, typical number of cigarettes consumed per day if reported to be current smoker, frequency of drinking, frequency of farming, exercise frequency, self-reported health status, waist circumference (with 2 degrees of freedom natural cubic spline), and asset-based wealth index quartile. Solid red line is showing point estimate with lighter red shade showing corresponding 95% confidence interval. Grey points represent observations.

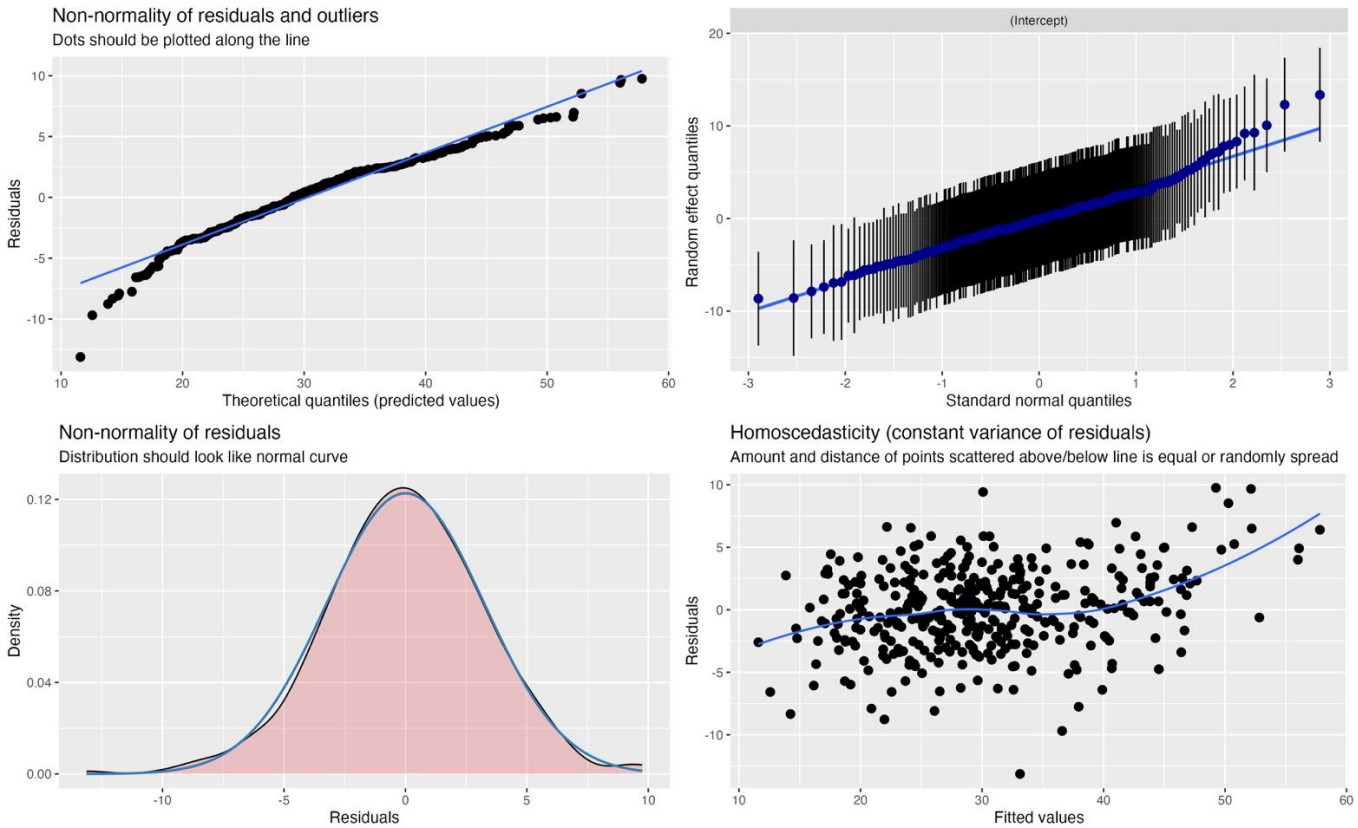

Figure S8. Diagnostic plots of the main multivariable mixed effects model for household $PM_{2.5}$. Note: The model is based on observed (non-imputed) complete cases data of 389 observations from 265 observations (i.e., no missing in wintertime household and outdoor $PM_{2.5}$, sex, age, marital status, highest education, current occupation, exposure to tobacco smoke, typical number of cigarettes consumed per day if reported to be current smoker, frequency of drinking, frequency of farming, exercise frequency, self-reported health status, waist circumference, and asset-based wealth index quartile). The multivariable model is adjusted with sex, age, marital status, highest education, current occupation, exposure to tobacco smoke, typical number of cigarettes consumed per day if reported to be current smoker, frequency of drinking, frequency of farming, exercise frequency, self-reported health status, waist circumference (with 2 degrees of freedom natural cubic spline), and asset-based wealth index quartile.

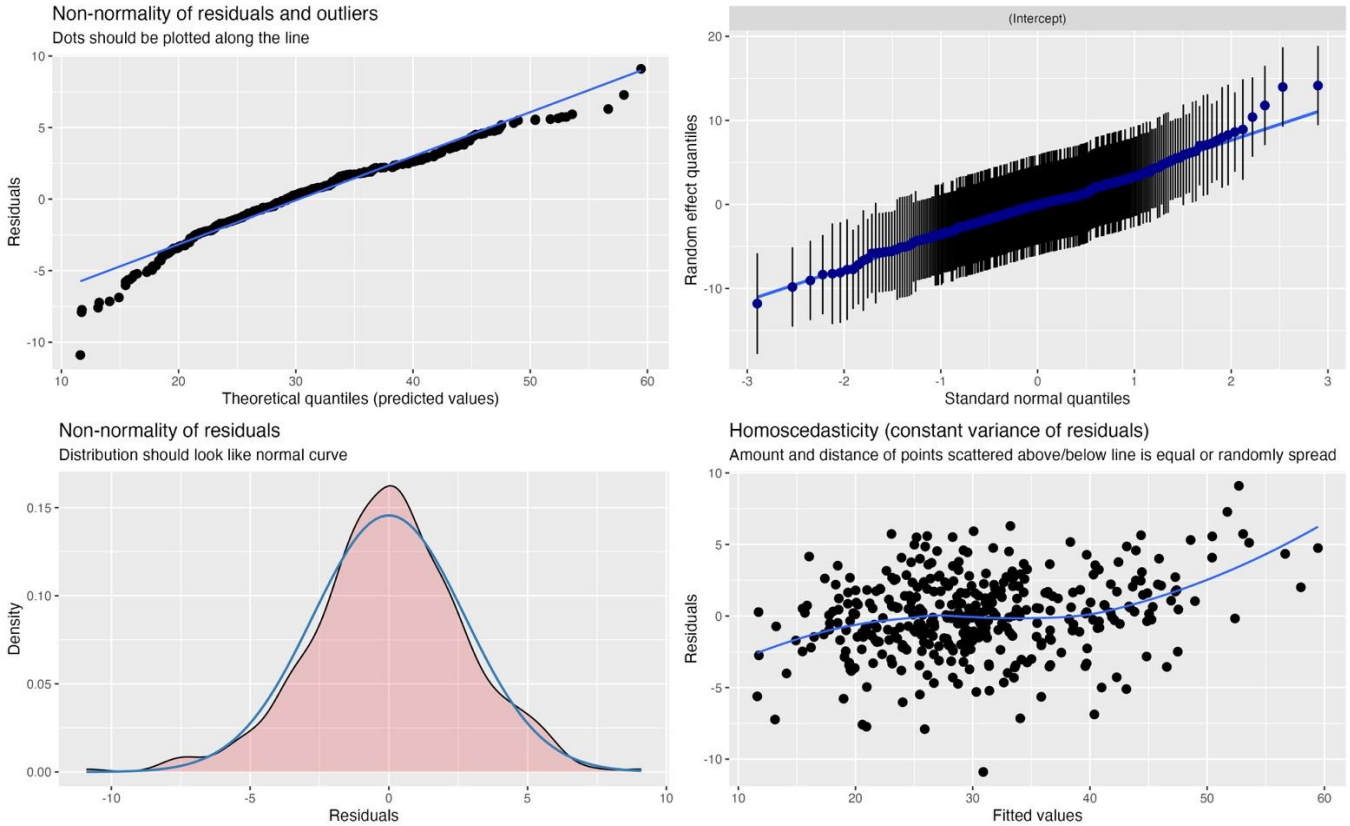

Figure S9. Diagnostic plots of the main multivariable mixed effects model for outdoor $PM_{2.5}$.

Note: The model is based on observed (non-imputed) complete cases data of 389 observations from 265 observations (i.e., no missing in wintertime household and outdoor $PM_{2.5}$, sex, age, marital status, highest education, current occupation, exposure to tobacco smoke, typical number of cigarettes consumed per day if reported to be current smoker, frequency of drinking, frequency of farming, exercise frequency, self-reported health status, waist circumference, and asset-based wealth index quartile). The multivariable model is adjusted with sex, age, marital status, highest education, current occupation, exposure to tobacco smoke, typical number of cigarettes consumed per day if reported to be current smoker, frequency of drinking, frequency of farming, exercise frequency, self-reported health status, waist circumference (with 2 degrees of freedom natural cubic spline), and asset-based wealth index quartile.

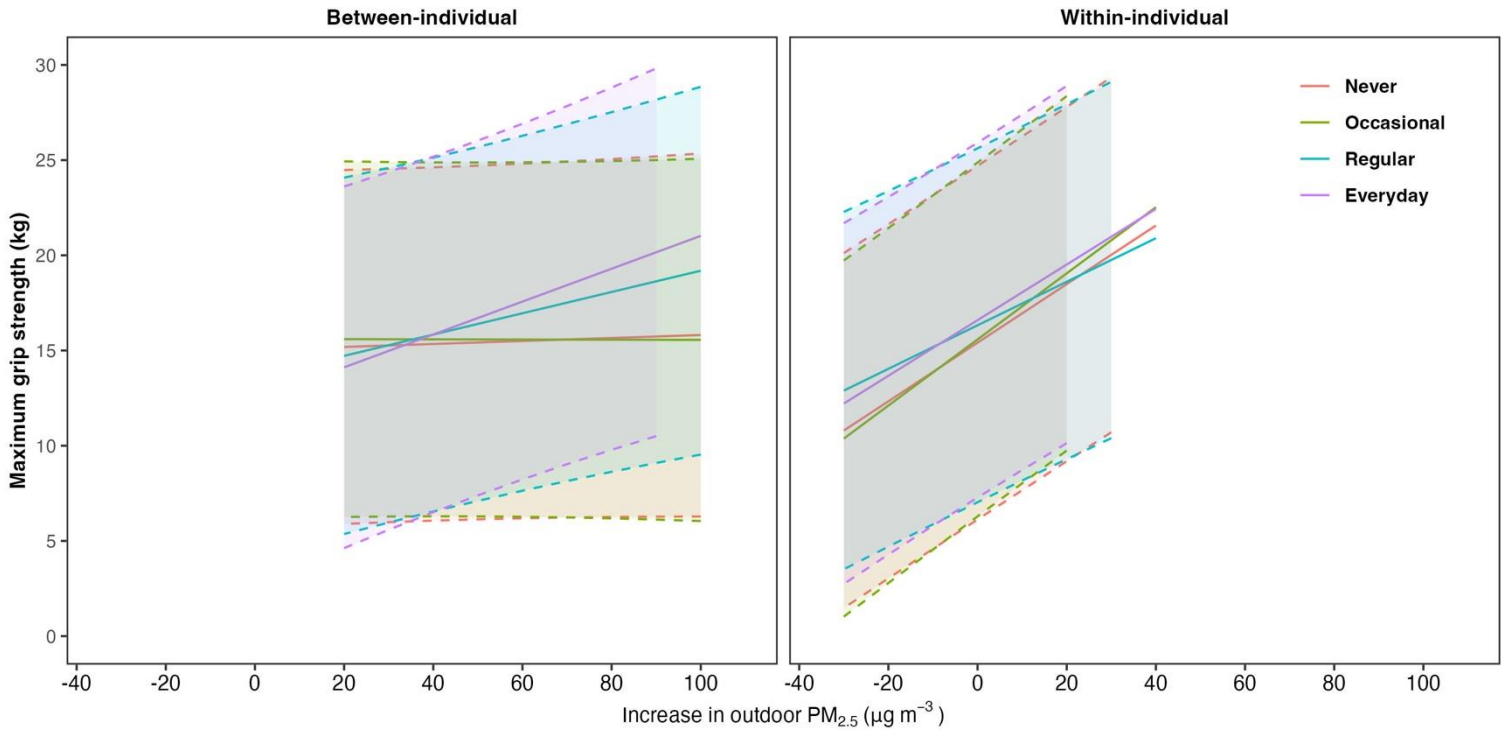

Figure S10. Marginal effects of outdoor $PM_{2.5}$ on maximum grip strength by frequency of farming. Note: The predictions are based on the main multivariable mixed effects model, adjusted with sex, age, marital status, highest education, current occupation, exposure to tobacco smoke, typical number of cigarettes consumed per day if reported to be current smoker, frequency of drinking, frequency of farming, exercise frequency, self-reported health status, waist circumference (with 2 degrees of freedom natural cubic spline), and asset-based wealth index quartile. Results are presented with point estimate (solid line) and corresponding 95% CI (dashed line).

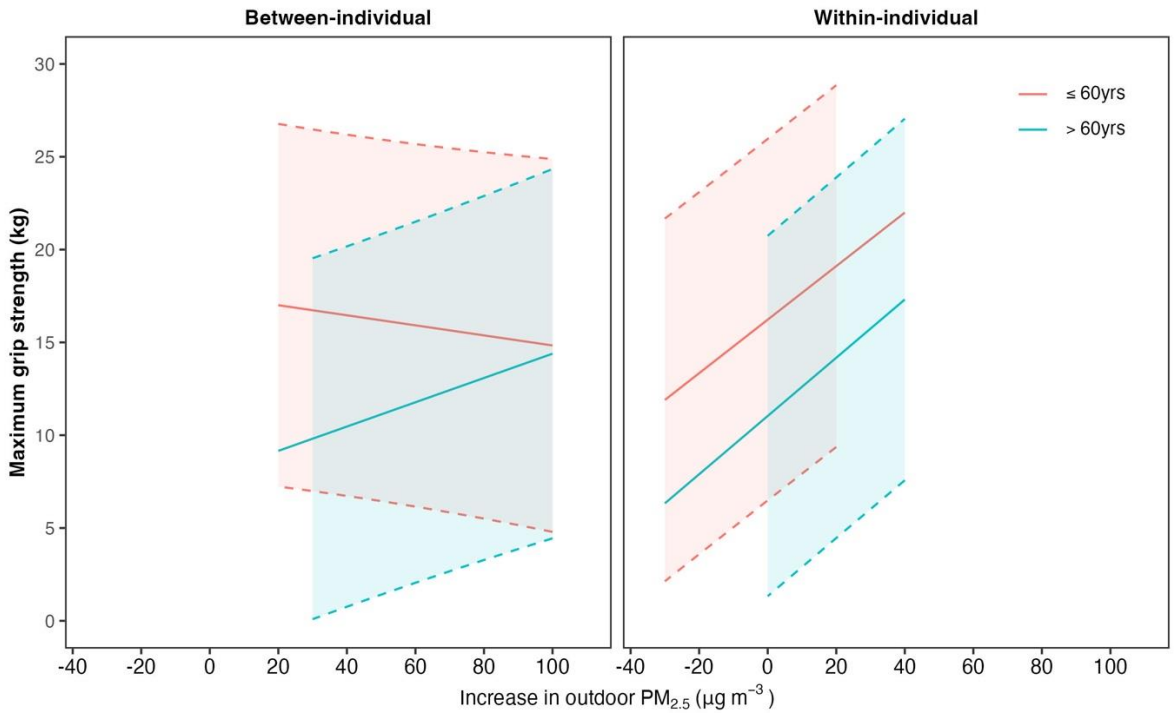

Figure S11. Marginal effects of outdoor $PM_{2.5}$ on maximum grip strength by age group. Note: The predictions are based on the main multivariable mixed effects model, adjusted with sex, age, marital status, highest education, current occupation, exposure to tobacco smoke, typical number of cigarettes consumed per day if reported to be current smoker, frequency of drinking, frequency of farming, exercise frequency, self-reported health status, waist circumference (with 2 degrees of freedom natural cubic spline), and asset-based wealth index quartile. Results are presented with point estimate (solid line) and corresponding 95% CI (dashed line).

Reference

33. Li, X.; Baumgartner, J.; Barrington-Leigh, C.; Harper, S.; Robinson, B.; Shen, G.; Sternbach, T.; Tao, S.; Zhang, X.; Zhang, Y.; et al. Socioeconomic and Demographic Associations with Wintertime Air Pollution Exposures at Household, Community, and District Scales in Rural Beijing, China. Environ. Sci. Technol. 2022, 56, 8308–8318. https://doi.org/10.1021/acs.est.1c07402

38. Rubin, D.B.; Schenker, N. Multiple Imputation in Health-Care Databases: An Overview and Some Applications. Stat. Med. 1991, 10, 585–598. https://doi.org/10.1002/sim.4780100410.
